# A Protocol for the Inclusion of Minoritized Persons in Alzheimer Disease Research From the ADNI3 Diversity Taskforce

**DOI:** 10.1001/jamanetworkopen.2024.27073

**Published:** 2024-08-09

**Authors:** Ozioma C. Okonkwo, Monica Rivera Mindt, Miriam T. Ashford, Catherine Conti, Joe Strong, Rema Raman, Michael C. Donohue, Rachel L. Nosheny, Derek Flenniken, Melanie J. Miller, Adam Diaz, Annabelle M. Soto, Beau M. Ances, Maryam R. Beigi, P. Murali Doraiswamy, Ranjan Duara, Martin R. Farlow, Hillel T. Grossman, Jacobo E. Mintzer, Christopher Reist, Emily J. Rogalski, Marwan N. Sabbagh, Stephen Salloway, Lon S. Schneider, Raj C. Shah, Ronald C. Petersen, Paul S. Aisen, Michael W. Weiner

**Affiliations:** 1 Department of Medicine and Wisconsin Alzheimer’s Disease Research Center, University of Wisconsin School of Medicine and Public Health, Madison; 2 Department of Psychology, Latin American Latinx Studies Institute, and African and African American Studies, Fordham University, Bronx, New York; 3 Department of Neurology, Icahn School of Medicine at Mount Sinai, New York, New York; 4 Northern California Institute for Research and Education, Department of Veterans Affairs Medical Center, San Francisco; 5 VA Advanced Imaging Research Center, San Francisco Veteran’s Administration Medical Center, San Francisco, California; 6 Alzheimer’s Therapeutic Research Institute, University of Southern California, San Diego; 7 Department of Psychiatry and Behavioral Sciences, University of California, San Francisco; 8 Department of Neurology, Washington University in Saint Louis, Saint Louis, Missouri; 9 Department of Neurology, David Geffen School of Medicine, University of California, Los Angeles; 10 Departments of Psychiatry and Medicine, Duke University School of Medicine, Durham, North Carolina; 11 Wein Center for Alzheimer’s Disease and Memory Disorders, Mount Sinai Medical Center, Miami Beach, Florida; 12 Herbert Wertheim College of Medicine, Florida International University, Miami; 13 Alzheimer’s Disease Research Center, University of Florida College of Medicine, Gainesville; 14 Department of Neurology, Indiana University Health, Indianapolis; 15 The Alzheimer Disease Research Center, Mount Sinai School of Medicine, New York, New York; 16 Medical University of South Carolina, Ralph H. Johnson VA Healthcare Center, Charleston; 17 MindX Sciences Inc, Indianapolis, Indiana; 18 Science 37 Inc, Durham, North Carolina; 19 Department of Psychiatry, University of California Irvine, Long Beach; 20 Department of Neurology, University of Chicago, Chicago, Illinois; 21 Alzheimer’s and Memory Disorders Division, Department of Neurology, Barrow Neurological Institute, Phoenix, Arizona; 22 Memory and Aging Program, Butler Hospital, Alpert Medical School, Brown University, Providence, Rhode Island; 23 Department of Psychiatry and Behavioral Sciences, Department of Neurology, Alzheimer’s Disease Research Center, Keck School of Medicine of USC, Los Angeles, California; 24 Department of Family and Preventive Medicine and the Rush Alzheimer’s Disease Center, Rush University Medical Center, Chicago, Illinois; 25 Alzheimer’s Disease Research Center, Mayo Clinic College of Medicine, Rochester, Minnesota; 26 Department of Radiology and Biomedical Imaging, University of California, San Francisco; 27 Department of Medicine, University of California, San Francisco; 28 Department of Neurology, University of California, San Francisco

## Abstract

**Importance:**

Black or African American (hereinafter, Black) and Hispanic or Latino/a/x (hereinafter, Latinx) adults are disproportionally affected by Alzheimer disease, but most research studies do not enroll adequate numbers of both of these populations. The Alzheimer’s Disease Neuroimaging Initiative-3 (ADNI3) launched a diversity taskforce to pilot a multipronged effort to increase the study inclusion of Black and Latinx older adults.

**Objective:**

To describe and evaluate the culturally informed and community-engaged inclusion efforts to increase the screening and enrollment of Black and Latinx older adults in ADNI3.

**Design, Setting, and Participants:**

This cross-sectional study used baseline data from a longitudinal, multisite, observational study conducted from January 15, 2021, to July 12, 2022, with no follow-up. The study was conducted at 13 ADNI3 sites in the US. Participants included individuals aged 55 to 90 years without cognitive impairment and those with mild cognitive impairment or Alzheimer disease.

**Exposures:**

Efforts included (1) launch of an external advisory board, (2) changes to the study protocol, (3) updates to the digital prescreener, (4) selection and deployment of 13 community-engaged research study sites, (5) development and deployment of local and centralized outreach efforts, and (6) development of a community-science partnership board.

**Main Outcomes and Measures:**

Screening and enrollment numbers from centralized and local outreach efforts, digital advertisement metrics, and digital prescreener completion.

**Results:**

A total of 91 participants enrolled in the trial via centralized and local outreach efforts, of which 22 (24.2%) identified as Latinx and 55 (60.4%) identified as Black (median [IQR] age, 65.6 [IQR, 61.5-72.5] years; 62 women [68.1%]). This represented a 267.6% increase in the monthly rate of enrollment (before: 1.11 per month; during: 4.08 per month) of underrepresented populations. For the centralized effort, social media advertisements were run between June 1, 2021, and July 31, 2022, which resulted in 2079 completed digital prescreeners, of which 1289 met criteria for subsequent site-level screening. Local efforts were run between June 1, 2021, to July 31, 2022. A total of 151 participants underwent site-level screening (100 from local efforts, 41 from centralized efforts, 10 from other sources).

**Conclusions and Relevance:**

In this cross-sectional study of pilot inclusion efforts, a culturally informed, community-engaged approach increased the inclusion of Black and Latinx participants in an Alzheimer disease cohort study.

## Key Points

**Question:**

Can multipronged culturally informed inclusion efforts increase the screening and enrollment of Black and Latinx older adults into Alzheimer’s Disease Neuroimaging Initiative-3?

**Findings:**

In this cross-sectional study of 1289 individuals who responded to centralized and local outreach efforts and underwent successful prescreening for the study, 91 participants enrolled, of which 86 were from underrepresented communities. This multipronged culturally informed inclusion pilot effort was associated with a 268% increase in the monthly rate of enrollment of underrepresented communities.

**Meaning:**

The findings provide initial evidence supporting a culturally informed community-engaged approach for increasing inclusion of Black and Latinx older adults in Alzheimer disease cohort studies.

## Teaser

This cross-sectional study examines the use of community-based efforts to increase the level of screening and enrollment of Black and Latinx individuals into Alzheimer disease studies through the Alzheimer’s Disease Neuroimaging Initiative-3 (ADNI3).

## Introduction

Black or African American (hereinafter, Black) and Hispanic or Latinx/a/o (hereinafter, Latinx) older adults are more likely to develop Alzheimer disease (AD) and other dementias compared with non-Latinx White older adults.^1,2,3^ With some exceptions,^4,5,6,7,8,9,10,11,12,13,14,15^ most AD research has not created conditions that allow for equitable inclusion of populations most affected by AD inequities.^4,16,17,18,19,20,21,22,23,24,25,26,27,28,29,30,31,32,33^ Underinclusion has serious ethical and scientific implications, including limiting the external and internal validity of research, that perpetuate AD-related inequities. Underinclusion leads to a lack of generalizability of treatment trials, which may seriously affect the risks and benefits of approved treatments in underrepresented populations (URPs). Therefore, it is necessary to develop and evaluate approaches that will improve inclusion of URPs into AD research studies.

Community-engaged research (CER) is an evidence-based approach to improve the inclusion and engagement of URPs into clinical research.^31,34^ In CER, study teams work iteratively, collaboratively, and in equitable partnership with community-based organizations, community stakeholders, and community members. Researchers build and sustain relationships through co-learning and power sharing, earning and maintaining the trust of the community, and prioritizing giving back to the community first. This evidence-based approach has been underused by many AD investigators^31,35,36^ and has been primarily deployed in local in-person settings.

Like many other studies in the field, the Alzheimer’s Disease Neuroimaging Initiative (ADNI) historically has not equitably enrolled URPs.^28,37^ To address this issue, the ADNI3 Diversity Taskforce (DVTF) was established to deploy a culturally informed CER pilot study for increasing the inclusion of Black and Latinx older adults into ADNI3. Preliminary findings have been published.^38^ Herein we aim to more fully describe the efforts deployed as part of this pilot study and detail our results, including local and centralized outreach efforts, completion of a digital prescreener, full site-level screening and enrollment metrics, and characteristics of enrolled participants.

## Methods

This study followed the Strengthening the Reporting of Observational Studies in Epidemiology (STROBE) reporting guideline. The ADNI3 was approved by the Advarra Independent Review Board. A signed informed consent form was obtained from all participants. Participants received financial compensation.

### Description of Diversity Taskforce Efforts

**Figure 1.**
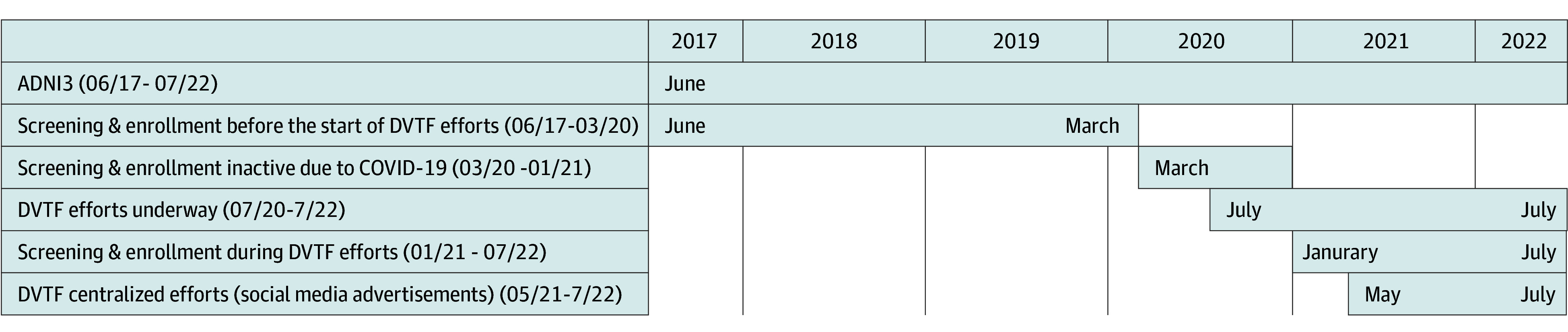
Timeline of Alzheimer’s Disease Neuroimaging Initiative-3 (ADNI3) and Diversity Taskforce (DVTF) Efforts

The ADNI3 DVTF was launched on July 1, 2020, and concluded July 31, 2022, at the end of the ADNI3 funding cycle (eMethods 1 in Supplement 1 provides a brief description of the ADNI). It was led by 2 co-principal investigators (O.C.O. and M.R.M.) with expertise in brain health inequities and was established to (1) develop, implement, and assess pilot efforts to increase inclusion and engagement of older Black and Latinx adults; (2) publish ADNI data related to diversity and health inequities; and (3) generate health inequity aims for ADNI4. Multipronged efforts included (1) development and launch of the external advisory board (EAB) (eMethods 2 in Supplement 1), (2) changes to the ADNI3 protocol (eMethods 3 in Supplement 1), (3) modifications to the existing digital prescreener form, (4) selection and deployment of 13 ADNI3 sites to serve as DVTF sites (eMethods 4 in Supplement 1), (5) development and deployment of culturally informed local and centralized outreach efforts (eFigure 1 in Supplement 1 provides an overview of DVTF inclusion pathways and efforts), and (6) establishment of the ADNI Community-Science Partnership Board (CSPB) (eMethods 5 in Supplement 1). Figure 1 shows a timeline of DVTF events. All materials for this effort were developed only in the English language. Information about ADNI3 study inclusion efforts before the DVTF efforts are included in eMethods 6 in Supplement 1.

### Local Efforts

Local DVTF efforts were site-led and focused on in-person outreach and included some remote efforts. Local efforts were run between June 1, 2021, and July 31, 2022. The sites scaled up their efforts depending on their site demographic characteristics and prior experience. eTable 1 in Supplement 1 lists the 13 DVTF sites and principal investigators. The sites were asked to provide a brief summary about types of community-based organizations and the types and formats of outreach events.

### Centralized Efforts

Centralized DVTF efforts focused on digital outreach. With feedback from the EAB, the DVTF commissioned a Latina-owned and -operated marketing agency experienced with the engagement of URPs to create and launch culturally informed digital outreach efforts for each site. Efforts included site-specific social media advertisements and study inclusion micro-websites, which are site-specific webpages that appear after a potential participant clicks on the social media advertisement.

#### Social Media Efforts

Paid social media efforts were deployed on Facebook and Instagram between June 1, 2021, and July 31, 2022. eTable 2 in Supplement 1 reports the social media performance metrics and their respective definitions obtained from Facebook Analytics.

The advertisements were tailored to each site’s location and goals (eg, based on the predominant ethnocultural identity in the site’s proximity). The messaging and imagery were aligned with cultural values and beliefs that appealed to the respective URP groups and was developed and informed by the expertise of the EAB, investigators, and marketing agency. The advertisements considered the audience’s age, location, and interests (eg, music). Social media advertising techniques included remarketing (eg, when a visitor who has already interacted with online digital efforts is served an advertisement) and look-alike audiences (ie, advertisements are served to social media users who share similar characteristics with users who have previously interacted with those advertisements). Unique hyperlinks with urchin tracking modules to a site-specific microsite allowed data linkage from the advertisement to the digital prescreener.

#### Study Inclusion Micro-Websites

Individuals clicking social media advertisements were directed to site-specific micro-websites. Depending on site needs and demographic characteristics, up to 2 micro-websites focusing on Black and/or Latinx communities were created (total of 20 unique micro-websites). Seven of the 13 sites had both Black- and Latinx-tailored micro-websites (n = 14), 5 had only Black-tailored micro-websites, and 1 had only a Latinx-tailored micro-website. The micro-websites were vetted by the study team and EAB. They included local branding and images designed to appeal to each site’s URPs (images and language), as well as a link to a digital prescreener.

### Digital Prescreener

**Figure 2.**
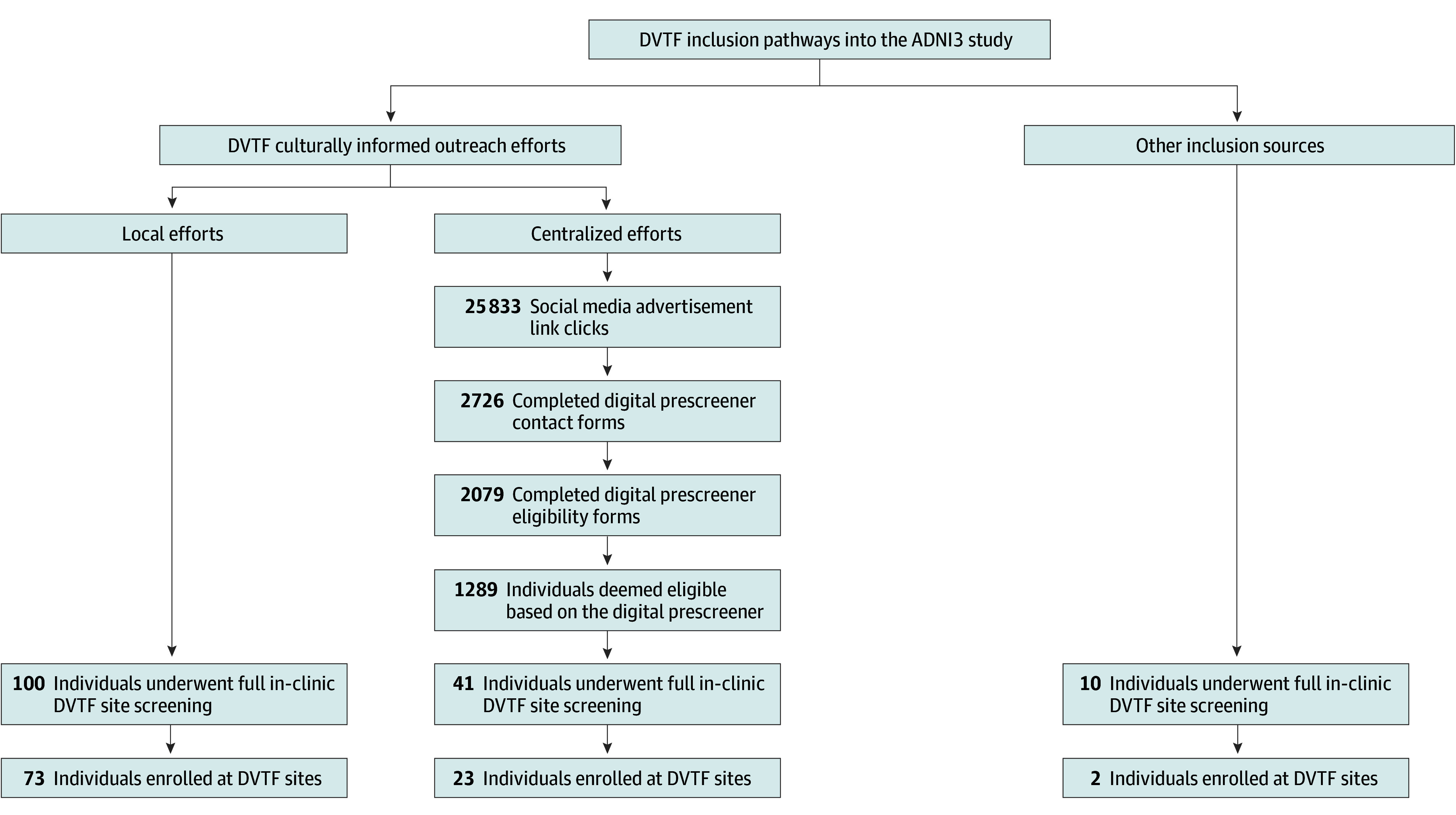
Flow Diagram of the Alzheimer’s Disease Neuroimaging Initiative-3 (ADNI3) Diversity Taskforce (DVTF) Efforts Digital Prescreener

Individuals who accessed the micro-websites were directed to complete a digital prescreener. The form was kept brief by asking only the most essential eligibility questions, with the aim of lowering potential barriers to study entry. It contained 2 main sections: contact form and eligibility form (Figure 2 shows prescreener flow and definitions). The information from completed prescreens was forwarded to the respective sites for further screening into ADNI3 or other studies. We captured the (1) number of completed contact forms in total and by microsite (Black- vs Latinx-tailored vs unknown, eg, instances where privacy settings scrubbed the urchin tracking modules from the URL), (2) number of completed eligibility forms, and (3) number of individuals deemed potentially eligible (meeting all eligibility criteria) (Figure 2), as well as the most common reasons for being deemed ineligible (multiple possible).

### Site-Level Screening and Enrollment Metrics

We report screening and enrollment data from 2 sources, each of which captured unique information. The first was DVTF site reports. Available data included the number of site-level screened and enrolled participants by outreach source (local vs centralized). The second was the ADNI database at the University of Southern California Laboratory of NeuroImaging (LONI). Available data included the number of site-level screened individuals and those whose screening was unsuccessful, the overall and monthly URP enrollment, and characteristics of enrolled participants (eMethods 7 in Supplement 1 provides information about self-reported participant characteristics).

Data regarding screening and enrollment provided directly by the DVTF sites do not fully match those obtained from the LONI database. This discrepancy is not entirely unusual, as it occurs across all of ADNI and is partly due to the usual time lag between study visits and fully curated data entry in the LONI. In the future, greater effort would be made to reduce such discrepancies.

### Statistical Analysis

Total URP screening frequencies and screen fail rates before and during DVTF efforts are reported. Total frequencies of advertisement metrics were tabulated for social media ads tailored to Black and Latinx adults. The Kruskal-Wallis test was used for continuous variables and Pearson χ^2^ test was conducted for categorical variables to evaluate differences in characteristics of participants enrolled before vs during the DVTF efforts. With 2-sided testing, the α level was set at .05, and *P* values are provided without adjustment for multiplicity. All analyses were conducted using R, version 4.2 (R Foundation for Statistical Computing).^39^

## Results

### Participant Characteristics

A total of 91 participants enrolled in the trial via centralized and local outreach efforts. The median age of the enrolled participants was 65.6 (IQR, 61.5-72.5) years, 62 (68.1%) identified as female, 29 (31.9%) as male, 22 (24.2%) as Latinx, 8 (8.8%) as non-Latinx Asian, 55 (60.4%) as non-Latinx Black, 5 (5.5%) as non-Latinx White, and 1 (1.1%) as non-Latinx Other (from other ethnocultural groups). Compared with those enrolled before the DVTF efforts, participants enrolled during the efforts were younger (71.8 vs 65.6 years; *P* < .001) and included a higher percentage of Latinx (7.9% vs 24.2%) and non-Latinx Black (6.1% vs 60.4%) (*P* < .001) individuals (Table 1).

**Table 1.** Characteristics of Participants Enrolled at DVTF Sites Before and During the DVTF Efforts^a^

Characteristic	Participants, No. (%)			*P* value
	Total enrolled (N = 256)	Enrolled before DVTF efforts [06/17-03/20] (n = 165)	Enrolled during DVTF efforts [01/21-07/22] (n = 91)	
Age, median (IQR), y	70.0 (65.3-76.3)	71.8 (67.1-77.5)	65.6 (61.5-72.5)	<.001^b^
Sex at birth				
Female	158 (61.)	96 (58.2)	62 (68.1)	.12^c^
Male	98 (38.3)	69 (41.8)	29 (31.9)	
Ethnocultural identity				
Latinx	35 (13.7)	13 (7.9)	22 (24.2)	<.001^c^
Non-Latinx Asian	18 (7.0)	10 (6.1)	8 (8.8)	
Non-Latinx Black	65 (25.4)	10 (6.1)	55 (60.4)	
Non-Latinx White	134 (52.3)	129 (78.2)	5 (5.5)	
Other non-Latinx^d^	4 (1.6)	3 (1.8)	1 (1.1)	
Education, median (IQR), y	16.0 (14.0-18.0)	16.0 (14.0-18.0)	16.0 (15.0-18.0)	.66^b^
Educational attainment group				
≤12 y	26 (10.2)	19 (11.5)	7 (7.7)	.33^c^
>12 y	230 (89.8)	146 (88.5)	84 (92.3)	
Diagnosis group				
AD	22 (8.6)	17 (10.3)	5 (5.5)	.27^c^
CU	160 (62.5)	98 (59.4)	62 (68.1)	
MCI	74 (28.9)	50 (30.3)	24 (26.4)	
Family history of AD/dementia				
Yes	143 (56.5)	97 (59.5)	46 (51.1)	.20^c^
No	110 (43.5)	66 (40.5)	44 (48.9)	
Missing	3	2	1	

Abbreviations: AD, Alzheimer disease; CU, cognitively unimpaired; DVTF, Diversity Taskforce; MCI, mild cognitive impairment.

^a^ Data are based on the University of Southern California Laboratory of NeuroImaging.

^b^ *P* values were determined using Kruskal-Wallis test.

^c^ *P* values were determined using Pearson χ^2^ test.

^d^ Includes individuals self-reporting their ethnicity as not Hispanic/Latino and as either American Indian/Alaska Native, Native Hawaiian/Pacific Islander, or individuals who indicated more than 1 race or declined to answer.

### Local Efforts

Based on the available data, local efforts used different types of community-based organizations and engaged in different types of outreach efforts (eTable 3 in Supplement 1 provides counts). The 2 most common types of community-based organizations were senior centers and local clinics. In-person outreach efforts included presentations, booths, and presence at community-based organization events, celebrations, and health fairs. Other in-person efforts included support groups and caregiver training. The 2 most common remote outreach efforts were engagement on local radio and television and webinars via a video conference.

### Centralized Efforts

A total of 198 advertisements were deployed, which reached 795 715 users (Table 2). There were 6771 reactions, 419 comments, 657 shares, and 226 saves. The links from the advertisements were clicked 25 833 times (1.1% click-through rate).

**Table 2.** Social Media Campaign Metric Results

Metric	Advertisements, No.		
	Total (N = 198)	Tailored to Black adults (n = 113)	Tailored to Latinx adults (n = 85)
Amount spent, $	124 731.63	72 075.18	52 656.45
Impressions	2 398 149	1 287 252	1 110 897
Reach	795 715	410 404	385 311
Link clicks	25 833	14 340	11 493
Click-through rate, %	1.1	1.1	1.0
Reactions	6771	3884	2887
Comments	419	176	243
Shares	657	425	232
Saves	226	130	96

### Digital Prescreening

**Figure 3.**
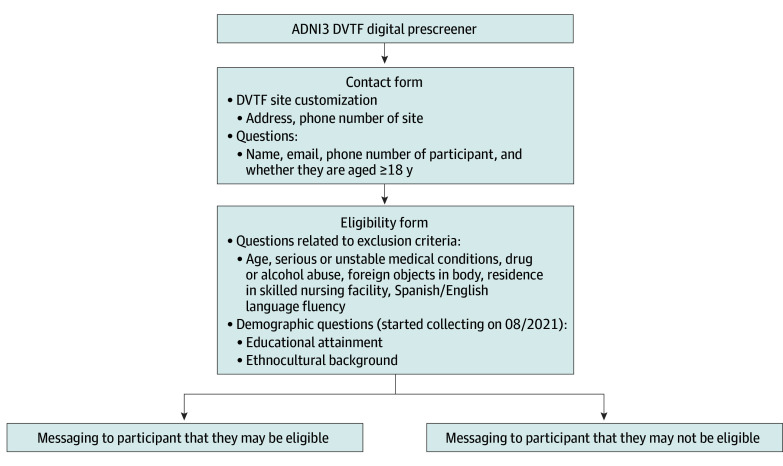
Results From Local and Centralized Outreach Efforts ADNI3 indicates Alzheimer’s Disease Neuroimaging Initiative-3; DVTF, Diversity Taskforce.

Link clicks from the digital advertisements resulted in 2726 completed contact forms (Figure 3). Of those, tracking identified that 688 forms (25.2%) were from Black-tailored micro-websites, 614 (22.5%) were from Latinx-tailored micro-websites, and 1424 (52.2%) were from unknown sources. A total of 2079 participants went on to complete the prescreener form. Of those who completed the screener form and reported race and ethnicity (n = 829), 425 individuals (51.3%) self-identified as Black, 145 (17.5%) self-identified as Latinx, and 95 (11.5%) self-identified as from other ethnocultural groups. Of the 2079 who completed the digital prescreener form, 1289 (62.0%) were deemed potentially eligible for ADNI. Among the 790 who were deemed potentially ineligible, the 3 most common reasons for ineligibility were (1) self-report of a serious or unstable medical illness diagnosis other than AD (n = 427), (2) not being aged 55 to 90 years (n = 300), and (3) having a pacemaker, other implanted medical device, metal fragments, or other foreign objects in the body (n = 289).

### Site-Level Screening and Enrollment

#### Screening Data Reported by the DVTF Sites

According to data reported by DVTF sites, 151 participants were site-level screened (Figure 3). Of those, 100 (66.2%) came from local efforts, 41 (27.2%) from centralized digital efforts, and 10 (6.6%) from other sources (eg, participant referral, web search).

#### Screening Data According to the LONI Database

According to the data in the LONI database, before the DVTF efforts (June 19, 2017, to March 9, 2020), the DVTF sites screened 237 participants (approximately 7 per month), with 50 (21.1%) URPs. Thirteen of the 50 individuals were excluded after the screening, resulting in a screen exclusion rate of 26.0%. During the DVTF efforts (June 15, 2021, to July 12, 2022), 145 individuals were screened (approximately 8 per month), of which 128 (88.3%) were URPs. Twenty of the 128 URPs were excluded after the screening, resulting in a screen exclusion rate of 15.6%. Among the 108 URPs whose screening was successful, 18 did not enroll. eFigure 2 in Supplement 1 provides a flowchart of the screening and enrollment numbers.

#### Enrollment Data Reported by the DVTF Sites

The DVTF sites reported enrolling a total of 98 participants (Figure 3). Of those, 73 (74.5%) came from local efforts, 23 (23.5%) from centralized efforts, and 2 (2.0%) from other sources.

#### Enrollment Data According to the LONI Database

According to the LONI (eFigure 2 in Supplement 1), 96 participants (90 URPs [93.8%]) initially enrolled. Five discontinued their participation early, of which 4 were URPs. This resulted in 91 enrolled participants with available demographic data, of which 86 (94.5%) were URPs (Table 2). The rate of enrollment of URPs improved from 1.11 per month before to 4.08 during the DVTF efforts, a 267.6% increase.

## Discussion

### Major Findings

Overall, this cross-sectional study found that multipronged and culturally informed CER-based outreach efforts are a feasible and robust way of increasing the representation of URPs in an ongoing observational AD study.

The first main finding was that local (in-person) and centralized (digital/social media) CER-based efforts were successful in connecting with individuals from Black and Latinx populations. This is evidenced by engagement with social media advertisements and micro-websites, prescreener completions, reduced URP screening exclusion rates, and the number of Black and Latinx participants who enrolled at sites. Despite a similar overall rate of participants screened before (approximately 7 per month) and during (approximately 8 per month) DVTF efforts, the DVTF efforts resulted in a higher percentage of URPs screened (50 of 237 individuals [21.1%] vs 128 of 145 individuals [88.3%]). The efforts were especially successful in increasing the percentage of enrolled Black participants. These findings add to the emerging literature related to in-person and digital CER-based outreach efforts as tools for increasing the engagement of URP older adults in AD research.^40,41,42,43,44,45^ In addition, the digital CER approach yielded comparable results in terms of social media metrics, for example, the click-through rate of our efforts (1.1%) is similar to the results of culturally informed social media inclusion efforts into an online registry (Brain Health Registry: 1.1%^44^). The digital CER approach allows for high scalability, but whether the approach is also feasible and efficacious in other URPs remains to be determined.

The second main finding was that local efforts resulted in more site-level screening and enrollment compared with centralized efforts. This highlights the crucial role of intensive local site-led efforts in connecting with the community for clinical studies. A previous study^46^ comparing local and social media inclusion and engagement efforts in AD research also found that in-person efforts were more successful in recruiting Black participants. However, the new centralized efforts required time to set up, thus ran for a shorter time and may not have been able to gain the same amount of recognition in communities that ongoing local efforts may have previously established. Furthermore, with our available data we could not compare the cost-effectiveness of local vs centralized efforts. However, social media inclusion and engagement efforts are potentially a useful addition to local efforts. While it remains unclear why only 41 of 1289 participants who were digitally prescreened underwent site-level screening, potential reasons include difficulties reaching participants, limited site staff capacity for leveraging the large numbers of digital referrals (eg, efforts began during the pandemic, and it took time for many sites to scale back up in their capacity to conduct in-person visits), and staff turnover (eg, referral emails sent to outdated site staff email addresses or received by yet-to-be-trained staff). In addition, some participants were excluded by additional site screening before full screening. In sum, this calls attention to the need to work more closely with sites to understand their capacity for digital referrals, a more robust digital prescreening process, and improved data capture for additional site screening. Our findings highlight the critical role of local efforts and that centralized digital efforts should be seen as an adjunct and not a replacement.

### Lessons Learned

#### URP Leadership and Staff With Health Inequities Expertise

One strength of this study was that the DVTF co-leads (O.C.O. and M.R.M.) possessed expertise in brain health inequities, CER, inclusion science, and/or AD and dementia. Moreover, there was genuine support (money, time, and human capital) by the ADNI leadership to implement this resource-intensive effort and provide CER and cultural competence training to all stakeholders. These requisite experience, expertise, support, and training factors were imperative for both leadership and staff.

#### Support and Reciprocal Communication With Study Sites

As study sites often run multiple studies, establishing reciprocal communication and adequate support is crucial. The DVTF team had regular email and video conferences with the sites not only to provide support and training but also to address issues that arose (eg, referrals from the centralized efforts being sent to staff no longer working on the study). Communication is important to regularly gauge site capacity, which helps manage site workload and informs the budget for digital advertisement for respective sites. It is also necessary to regularly obtain feedback on processes from clinical sites and responsively adjust to issues.

#### Planning and Budgeting for CER Efforts Ahead of Time

There is a substantial amount of groundwork, resources, and cost associated with the development and implementation of CER outreach efforts. Researchers interested in similar efforts should consider this during the planning and budgeting of their grant proposals. It is important to carefully plan time and budget costs for hiring and training diverse scientists and staff, establishing and maintaining CSPBs, hiring a marketing agency, supporting community-based organizations, developing and running centralized and local outreach materials, and developing platforms that integrate data from multiple efforts. It is essential to approach this work in a proactive and well-resourced manner that spans beyond the cyclical nature of grant funding periods to support communities (eg, providing psychoeducation, AD resources, and feedback about biomarker results), which in turn helps increase trust and participation.

#### Robust Data Capture of Site Outreach Efforts

While efforts were made to collect outreach effort data from the sites, more robust data linkage would be needed to more fully assess which efforts resulted in an enrollment and why. For example, we were unable to connect certain outreach data (eg, whether participants were from local or centralized efforts) to participant data in LONI. Improved data capture should include detailed information on local and centralized efforts (including resources and costs) and prescreening, as well as the ability to link outreach and prescreening data to in-clinic data. However, additional staff and participant burden need to be considered.

### Limitations

This pilot study was designed as a demonstration project, with a short timeframe for set-up and execution, resulting in multiple limitations. Limitations arose from the cross-sectional and nonexperimental design. Future studies should use longitudinal designs to examine the association between efforts and engagement over time and a nuanced assessment of specific components and intensity levels needed to yield desired outcomes. It is also notable that this effort was conducted during a difficult and volatile time in the US (eg, COVID-19 pandemic and heightened awareness of structural racism). Our pilot efforts were also not set up to collect all data necessary to connect the costs and outcomes of different combinations of the multipronged approach to screening, reasons for ineligibility, enrollment, and baseline participation results. Information about the total cost of running the efforts is needed (eg, costs for creating and running the advertisements, commissioning a marketing agency, investigator/staff to consult with the marketing agency, and cost of creating an EAB and CSPB). The analysis focused on inclusion metrics (screening and enrollment) and future studies should also evaluate engagement metrics, such as the completion of study procedures. The study did also not collect any data on whether the DVTF sites continued their prior recruitment efforts during the DVTF efforts period; future studies should assess and account for this.

Multiple selection biases affect the generalizability of the findings. For the digital efforts, this includes a bias for individuals with access to technology, including the internet. Further, the digital advertisements were not formally evaluated to estimate which concepts and messaging resonated most with the communities. The DVTF efforts were guided by the EAB, which did not include lay community members. The EAB evolved into the CSPB, which was established at the end of ADNI3. The CSPB guides the ADNI4 efforts and includes lay community members to more fully implement the ADNI CER approach and increase the diversity of perspectives. Furthermore, the DVTF efforts focused on Black and Latinx adults without additional consideration of other intersectional categories, such as years of education and socioeconomic status. Therefore, our sample may not be representative of Black and Latinx individuals in the broader community, for example, in terms of years of education, socioeconomic status, and health literacy. The enrollment of Latinx participants may have been limited by both our lack of Spanish-language landing pages and the sites’ ability to fully support Spanish-speaking participants. In ADNI4, we are expanding to other ethnocultural groups (eg, American Indian or Alaska Native, Asian, and Native Hawaiian or Pacific Islander) and are also facilitating the capacity of sites to fully engage Spanish-speaking participants. We are additionally planning outreach to other URPs, such as people from lower socioeconomic backgrounds, sexual and gender minorities, and rural dwellers. Because the integration of the DVTF digital metrics into the existing ADNI3 data platform was limited, there was no systematic collection of prescreen data prior to the efforts, and no data were collected on how protocol changes affected URP enrollment. We recommend that future studies exercise diligence early in project planning to ensure that tracking of data and metrics is fully optimized. The study limitations notwithstanding, the success of this pilot effort provided the justification and impetus for the new Engagement Core in ADNI4^47^ which, as noted, is expanding CER efforts to all ADNI sites and to other URPs. In addition, the results might be applicable to inclusion efforts into non-AD research studies.

## Conclusions

In this cross-sectional study of pilot research inclusion efforts, we found initial evidence supporting a comprehensive multipronged culturally informed CER approach for increasing the inclusion of URPs into AD research. Much work remains to make AD research more inclusive, equitable, and valid. The new Engagement Core in ADNI4 is well-positioned to contribute to this effort by methodically leveraging the learnings from these pilot efforts.

## Notes

Supplement 1. **eMethods 1.** Brief Alzheimer’s Disease Neuroimaging Initiative Description **eMethods 2.** Alzheimer’s Disease Neuroimaging Initiative-3 Diversity Taskforce External Advisory Board **eMethods 3.** Changes to the Alzheimer’s Disease Neuroimaging Initiative-3 Protocol **eMethods 4.** Selection and Deployment of Diversity Taskforce Sites **eMethods 5.** Development of the Alzheimer’s Disease Neuroimaging Initiative Community-Science Partnership Board **eMethods 6.** ADNI3 Inclusion Efforts Before the DVTF Efforts **eMethods 7.** Participant Characteristics Metrics **eTable 1.** ADNI3 DVTF Sites and Principal Investigators **eTable 2.** Social Media Performance Metrics **eTable 3.** Counts of Types of Community-Based Organizations and Types of Outreach Efforts **eFigure 1.** Overview of the Diversity Taskforce Inclusion Pathways and Efforts **eFigure 2.** Diversity Taskforce Site Screening and Enrollment Flowchart
eReferences

Supplement 2.
Data Sharing Statement

## References

[1] Tang MX, Stern Y, Marder K, . The *APOE*-epsilon4 allele and the risk of Alzheimer disease among African Americans, Whites, and Hispanics. JAMA. 1998;279(10):751-755. doi:10.1001/jama.279.10.751 9508150

[2] Weuve J, Barnes LL, Mendes de Leon CF, . Cognitive aging in Black and White Americans: cognition, cognitive decline, and incidence of Alzheimer disease dementia. Epidemiology. 2018;29(1):151-159. doi:10.1097/EDE.0000000000000747 28863046 PMC5718953

[3] Alzheimer’s Association. Alzheimer’s disease facts and figures. 2024. Accessed July 24, 2024. https://www.alz.org/media/documents/alzheimers-facts-and-figures.pdf.

[4] Epps FR, Skemp L, Specht J. Using culturally informed strategies to enhance recruitment of African Americans in dementia research: a nurse researcher’s experience. J Res Pract. 2015;11(1):M2-M2.

[5] Hendrie HC, Hall KS, Hui S, . Apolipoprotein E genotypes and Alzheimer’s disease in a community study of elderly African Americans. Ann Neurol. 1995;37(1):118-120. doi:10.1002/ana.410370123 7818244

[6] Marquez DX, Glover CM, Lamar M, . Representation of Older Latinxs in Cohort Studies at the Rush Alzheimer’s Disease Center. Neuroepidemiology. 2020;54(5):404-418. doi:10.1159/000509626 32906123 PMC7572552

[7] Zeki Al Hazzouri A, Haan MN, Kalbfleisch JD, Galea S, Lisabeth LD, Aiello AE. Life-course socioeconomic position and incidence of dementia and cognitive impairment without dementia in older Mexican Americans: results from the Sacramento Area Latino Study on Aging. Am J Epidemiol. 2011;173(10):1148-1158. doi:10.1093/aje/kwq483 21430188 PMC3121319

[8] Martinez-Miller EE, Robinson WR, Avery CL, . Acculturation, cognitive performance and decline, and incident dementia/CIND: the Sacramento Area Latino Study on Aging. Am J Epidemiol. 2020;189(11):1292-1305. doi:10.1093/aje/kwaa088 32440686 PMC7604518

[9] González HM, Tarraf W, Gouskova N, . Neurocognitive function among middle-aged and older Hispanic/Latinos: results from the Hispanic Community Health Study/Study of Latinos. Arch Clin Neuropsychol. 2015;30(1):68-77. doi:10.1093/arclin/acu066 25451561 PMC4351363

[10] Mindt MR, Miranda C, Arentoft A, . Aging and HIV/AIDS: neurocognitive implications for older HIV-positive Latina/o adults. Behav Med. 2014;40(3):116-123. doi:10.1080/08964289.2014.914464 25090364 PMC5584638

[11] Barnes LL, Wilson RS, Li Y, Gilley DW, Bennett DA, Evans DA. Change in cognitive function in Alzheimer’s disease in African-American and White persons. Neuroepidemiology. 2006;26(1):16-22. doi:10.1159/000089231 16254449

[12] Barnes LL, Mendes de Leon CF, Wilson RS, Bienias JL, Evans DA. Social resources and cognitive decline in a population of older African Americans and Whites. Neurology. 2004;63(12):2322-2326. doi:10.1212/01.WNL.0000147473.04043.B3 15623694

[13] Lamar M, Durazo-Arvizu RA, Sachdeva S, . Cardiovascular disease risk factor burden and cognition: implications of ethnic diversity within the Hispanic Community Health Study/Study of Latinos. PLoS One. 2019;14(4):e0215378. doi:10.1371/journal.pone.0215378 31009492 PMC6476505

[14] Avila JF, Rentería MA, Jones RN, . Education differentially contributes to cognitive reserve across racial/ethnic groups. Alzheimers Dement. 2021;17(1):70-80. doi:10.1002/alz.12176 32827354 PMC8376080

[15] Roosa MW, Liu FF, Torres M, Gonzales NA, Knight GP, Saenz D. Sampling and recruitment in studies of cultural influences on adjustment: a case study with Mexican Americans. J Fam Psychol. 2008;22(2):293-302. doi:10.1037/0893-3200.22.2.293 18410216 PMC2730376

[16] Wong R, Amano T, Lin SY, Zhou Y, Morrow-Howell N. Strategies for the recruitment and retention of racial/ethnic minorities in Alzheimer disease and dementia clinical research. Curr Alzheimer Res. 2019;16(5):458-471. doi:10.2174/1567205016666190321161901 30907319

[17] Canevelli M, Bruno G, Grande G, . Race reporting and disparities in clinical trials on Alzheimer’s disease: a systematic review. Neurosci Biobehav Rev. 2019;101:122-128. doi:10.1016/j.neubiorev.2019.03.020 30946856

[18] Areán PA, Alvidrez J, Nery R, Estes C, Linkins K. Recruitment and retention of older minorities in mental health services research. Gerontologist. 2003;43(1):36-44. doi:10.1093/geront/43.1.36 12604744

[19] Bergeron CD, Foster C, Friedman DB, Tanner A, Kim SH. Clinical trial recruitment in rural South Carolina: a comparison of investigators’ perceptions and potential participant eligibility. Rural Remote Health. 2013;13(4):2567. doi:10.22605/RRH2567 24325179

[20] Chadiha LA, Washington OG, Lichtenberg PA, Green CR, Daniels KL, Jackson JS. Building a registry of research volunteers among older urban African Americans: recruitment processes and outcomes from a community-based partnership. Gerontologist. 2011;51(suppl 1)(suppl 1):S106-S115. doi:10.1093/geront/gnr034 21565812 PMC3092980

[21] Grill JD, Holbrook A, Pierce A, Hoang D, Gillen DL. Attitudes toward potential participant registries. J Alzheimers Dis. 2017;56(3):939-946. doi:10.3233/JAD-160873 28106553 PMC5533604

[22] McHenry JC, Insel KC, Einstein GO, Vidrine AN, Koerner KM, Morrow DG. Recruitment of older adults: success may be in the details. Gerontologist. 2015;55(5):845-853. doi:10.1093/geront/gns079 22899424 PMC4592329

[23] Glover CM, Creel-Bulos C, Patel LM, . Facilitators of research registry enrollment and potential variation by race and gender. J Clin Transl Sci. 2018;2(4):234-238. doi:10.1017/cts.2018.326 31660225 PMC6798442

[24] Jefferson AL, Lambe S, Chaisson C, Palmisano J, Horvath KJ, Karlawish J. Clinical research participation among aging adults enrolled in an Alzheimer’s Disease Center research registry. J Alzheimers Dis. 2011;23(3):443-452. doi:10.3233/JAD-2010-101536 21116048 PMC3178330

[25] Lee JY, Crooks RE, Pham T, . “If it helps someone, then I want to do it”: perspectives of persons living with dementia on research registry participation. Dementia (London). 2019;1471301219827709. 30722693 10.1177/1471301219827709

[26] Zhou Y, Elashoff D, Kremen S, Teng E, Karlawish J, Grill JD. African Americans are less likely to enroll in preclinical Alzheimer’s disease clinical trials. Alzheimers Dement (N Y). 2016;3(1):57-64. doi:10.1016/j.trci.2016.09.004 29067319 PMC5651355

[27] Auster J, Janda M. Recruiting older adults to health research studies: a systematic review. Australas J Ageing. 2009;28(3):149-151. doi:10.1111/j.1741-6612.2009.00362.x 19845656

[28] Birkenbihl C, Salimi Y, Domingo-Fernándéz D, Lovestone S, Fröhlich H, Hofmann-Apitius M; AddNeuroMed consortium; Japanese Alzheimer’s Disease Neuroimaging Initiative; and the Alzheimer’s Disease Neuroimaging Initiative. Evaluating the Alzheimer’s disease data landscape. Alzheimers Dement (N Y). 2020;6(1):e12102. doi:10.1002/trc2.12102 33344750 PMC7744022

[29] Fargo KN, Carrillo MC, Weiner MW, Potter WZ, Khachaturian Z. The crisis in recruitment for clinical trials in Alzheimer’s and dementia: an action plan for solutions. Alzheimers Dement. 2016;12(11):1113-1115. doi:10.1016/j.jalz.2016.10.001 27836052

[30] Shin J, Doraiswamy PM. Underrepresentation of African-Americans in Alzheimer’s trials: a call for affirmative action. Front Aging Neurosci. 2016;8:123. doi:10.3389/fnagi.2016.00123 27375473 PMC4891330

[31] Gilmore-Bykovskyi AL, Jin Y, Gleason C, . Recruitment and retention of underrepresented populations in Alzheimer’s disease research: a systematic review. Alzheimers Dement (N Y). 2019;5:751-770. doi:10.1016/j.trci.2019.09.018 31921966 PMC6944728

[32] Gleason CE, Zuelsdorff M, Gooding DC, . Alzheimer’s disease biomarkers in Black and non-Hispanic White cohorts: a contextualized review of the evidence. Alzheimers Dement. 2022;18(8):1545-1564. doi:10.1002/alz.12511 34870885 PMC9543531

[33] Franzen S, Smith JE, van den Berg E, . Diversity in Alzheimer’s disease drug trials: the importance of eligibility criteria. Alzheimers Dement. 2022;18(4):810-823. doi:10.1002/alz.12433 34590409 PMC8964823

[34] Wieland ML, Njeru JW, Alahdab F, Doubeni CA, Sia IG. Community-engaged approaches for minority recruitment into clinical research: a scoping review of the literature. Mayo Clin Proc. 2021;96(3):733-743. doi:10.1016/j.mayocp.2020.03.028 33004216

[35] Hinton L, Carter K, Reed BR, . Recruitment of a community-based cohort for research on diversity and risk of dementia. Alzheimer Dis Assoc Disord. 2010;24(3):234-241. doi:10.1097/WAD.0b013e3181c1ee01 20625273 PMC2946798

[36] Romero HR, Welsh-Bohmer KA, Gwyther LP, . Community engagement in diverse populations for Alzheimer disease prevention trials. Alzheimer Dis Assoc Disord. 2014;28(3):269-274. doi:10.1097/WAD.0000000000000029 24614272 PMC4139415

[37] Ashford MT, Raman R, Miller G, ; Alzheimer’s Disease Neuroimaging Initiative. Screening and enrollment of underrepresented ethnocultural and educational populations in the Alzheimer’s Disease Neuroimaging Initiative (ADNI). Alzheimers Dement. 2022;18(12):2603-2613. doi:10.1002/alz.12640 35213778 PMC9402812

[38] Rivera Mindt M, Okonkwo O, Weiner MW, . Improving generalizability and study design of Alzheimer’s disease cohort studies in the United States by including under-represented populations. Alzheimers Dement. 2023;19(4):1549-1557. 36372959 10.1002/alz.12823 PMC10101866

[39] Language and environment for statistical computing. R Foundation for Statistical Computing; 2017.

[40] Watson B, Robinson DH, Harker L, Arriola KRJ. The inclusion of African-American study participants in web-based research studies. J Med Internet Res. 2016;18(6):e168. doi:10.2196/jmir.5486 27334683 PMC4935793

[41] Cowie JM, Gurney ME. The use of Facebook advertising to recruit healthy elderly people for a clinical trial: baseline metrics. JMIR Res Protoc. 2018;7(1):e20. doi:10.2196/resprot.7918 29367186 PMC5803529

[42] Wozney L, Turner K, Rose-Davis B, McGrath PJ. Facebook ads to the rescue? recruiting a hard to reach population into an internet-based behavioral health intervention trial. Internet Interv. 2019;17:100246. doi:10.1016/j.invent.2019.100246 31080751 PMC6500917

[43] Bour C, Ahne A, Schmitz S, Perchoux C, Dessenne C, Fagherazzi G. The use of social media for health research purposes: scoping review. J Med Internet Res. 2021;23(5):e25736. doi:10.2196/25736 34042593 PMC8193478

[44] Ashford MT, Camacho MR, Jin C, . Digital culturally tailored marketing for enrolling Latino participants in a web-based registry: baseline metrics from the Brain Health Registry. Alzheimers Dement. 2023;19(5):1714-1728. doi:10.1002/alz.12805 36193827 PMC10070578

[45] Mindt MR, Ashford MT, Zhu D, . The Community Engaged Digital Alzheimer’s Research (CEDAR) Study: a digital intervention to increase research participation of Black American participants in the Brain Health Registry. J Prev Alzheimers Dis. 2023;10(4):847-856. doi:10.14283/jpad.2023.32 37874107 PMC10598330

[46] Stout SH, Babulal GM, Johnson AM, Williams MM, Roe CM. Recruitment of African American and non-Hispanic white older adults for Alzheimer disease research via traditional and social media: a case study. J Cross Cult Gerontol. 2020;35(3):329-339. doi:10.1007/s10823-020-09405-9 32712751 PMC7418860

[47] Weiner MW, Veitch DP, Miller MJ, . Increasing participant diversity in AD research: plans for digital screening, blood testing, and a community-engaged approach in the Alzheimer’s Disease Neuroimaging Initiative 4. Alzheimers Dement. 2023;19(1):307-317. doi:10.1002/alz.12797 36209495 PMC10042173

