# Supplementary material for: A Protocol for the Inclusion of Minoritized Persons in Alzheimer Disease Research From the ADNI3 Diversity Taskforce
Source: JAMA Netw Open. 2024 Aug 9;7(8):e2427073. doi: 10.1001/jamanetworkopen.2024.27073 (PMC11316236; doi:10.1001/jamanetworkopen.2024.27073)
Supplement: Supplement 1. — eMethods 1. Brief Alzheimer’s Disease Neuroimaging Initiative Description eMethods 2. Alzheimer’s Disease Neuroimaging Initiative-3 Diversity Taskforce External Advisory Board eMethods 3. Changes to the Alzheimer’s Disease Neuroimaging Initiative-3 Protocol eMethods 4. Selection and Deployment of Diversity Taskforce Sites eMethods 5. Development of the Alzheimer’s Disease Neuroimaging Initiative Community-Science Partnership Board eMethods 6. ADNI3 Inclusion Efforts Before the DVTF Efforts eMethods 7. Participant Characteristics Metrics eTable 1. ADNI3 DVTF Sites and Principal Investigators eTable 2. Social Media Performance Metrics eTable 3. Counts of Types of Community-Based Organizations and Types of Outreach Efforts eFigure 1. Overview of the Diversity Taskforce Inclusion Pathways and Efforts eFigure 2. Diversity Taskforce Site Screening and Enrollment Flowchart eReferences [file jamanetwopen-e2427073-s001.pdf]

Supplementary Online Content

Okonkwo OC, Rivera Mindt M, Ashford MT, et al. .A protocol for the inclusion of minoritized persons in Alzheimer disease research from the ADNI3 Diversity Taskforce. *JAMA Netw Open*. 2024;7(8):e2427073. doi:10.1001/jamanetworkopen.2024.27073

eMethods 1. Brief Alzheimer’s Disease Neuroimaging Initiative Description

eMethods 2. Alzheimer’s Disease Neuroimaging Initiative-3 Diversity Taskforce External Advisory Board

eMethods 3. Changes to the Alzheimer’s Disease Neuroimaging Initiative-3 Protocol

eMethods 4. Selection and Deployment of Diversity Taskforce Sites

eMethods 5. Development of the Alzheimer’s Disease Neuroimaging Initiative Community-Science Partnership Board

eMethods 6. ADNI3 Inclusion Efforts Before the DVTF Efforts

eMethods 7. Participant Characteristics Metrics

eTable 1. ADNI3 DVTF Sites and Principal Investigators

eTable 2. Social Media Performance Metrics

eTable 3. Counts of Types of Community-Based Organizations and Types of Outreach Efforts

eFigure 1. Overview of the Diversity Taskforce Inclusion Pathways and Efforts

eFigure 2. Diversity Taskforce Site Screening and Enrollment Flowchart

eReferences

This supplementary material has been provided by the authors to give readers additional information about their work.

© 2024 Okonkwo OC et al. *JAMA Network Open.*

eMethods 1. Brief Alzheimer's Disease Neuroimaging Initiative Description

The Alzheimer's Disease Neuroimaging Initiative (ADNI) was launched in 2003 as a public-private partnership, led by Principal Investigator Michael W. Weiner, MD. ADNI is an ongoing, multicenter, longitudinal study which aims to develop and validate clinical, imaging, genetic, and biochemical biomarkers for AD clinical trials [1-4]. ADNI enrolls participants aged 55 to 90 who are either classified as cognitively unimpaired, having mild cognitive impairment or dementia due to AD. So far, there have been five phases (ADNI1, ADNI-GO, ADNI2, ADNI3, ADNI4). The efforts described in this paper were implemented during the ADNI3 phase.

eMethods 2. Alzheimer's Disease Neuroimaging Initiative-3 Diversity Taskforce External Advisory Board

To help guide the Diversity Taskforce (DVTF) efforts, a six-member Alzheimer's Disease Neuroimaging Initiative-3 (ADNI3) DVTF External Advisory Board (EAB) was formed in Fall 2020, comprised of scientists with expertise in health equity research (please see Acknowledgements for the complete list). Through a highly collaborative and iterative process, the EAB played a key role in guiding the DVTF and ensuring that study materials and methods were culturally-informed and based upon CER principles. To this end, the EAB convened virtual biannual meetings with the DVTF Co-Leads and team, beginning in November 2020. During these meetings, DVTF progress and strategies were presented, and EAB members offered questions, constructive feedback, and next-step recommendations. In addition, EAB members generously provided periodic consultation, troubleshooting, and support (via phone and email) between these meetings. The EAB concluded in July 2022 with the launch of ADNI's ongoing Community-Science Partnership Board (detailed in eMethods 5).

eMethods 3. Changes to the Alzheimer's Disease Neuroimaging Initiative-3 protocol

Based on the recommendations of the Diversity Taskforce (DVTF) leadership and the EAB, and with the approval of Alzheimer's Disease Neuroimaging Initiative-3's (ADNI3) leadership and relevant site PIs, ad hoc protocol changes were piloted at DVTF study sites to facilitate research participation of persons from under-included communities. The changes included: providing Aβ PET result disclosure on an ad hoc basis by study site clinicians to participants who were interested in receiving their results; making lumbar puncture procedures optional; creating a remote study visit protocol during the COVID-19 pandemic to allay health and safety concerns; and loosening study entry criteria (e.g., study partner requirement, vascular comorbidities).

© 2024 Okonkwo OC et al. *JAMA Network Open.*

eMethods 4. Selection and deployment of Diversity Taskforce sites

eTable 1 summarizes the 13 Diversity Taskforce (DVTF) sites from across the U.S. Selected sites received funding and support, including: $150,000 each per year for staff and activities; training, coaching, and mentorship in community-engaged research (CER) and cultural competence methods; quarterly check-in meetings with the DVTF leadership for updates; and ad hoc consultations with DVTF leadership for troubleshooting and support. Two crucial ingredients of the DVTF's CER model include: (1) In addition to asking participants to join the ADNI study ("get"), efforts also included an emphasis on the "give" to the participants (e.g., providing participants with PET amyloid feedback) and the DVTF site (e.g., funding for outreach efforts). CER efforts, both local and centralized digital, were not simply meant to recruit participants into ADNI. Instead, these efforts focused on engaging communities that are typically under-included in research, with the goal of presenting the Alzheimer's Disease Neuroimaging Initiative (ADNI) as an organization that gives back to the community and to raise awareness that studies like ADNI (with this new CER focus, resources, and commitment) exist. (2) Strong support of, engagement in, and integration of the ADNI3 Principal Investigator (PI), Core leaders, and Site PIs into efforts. In consultation with our EAB, both local (i.e., "boots on the ground") and centralized (digital only) outreach efforts were deployed. See eFigure 1 for an overview of efforts.

© 2024 Okonkwo OC et al. *JAMA Network Open.*

eMethods 5. Development of the Alzheimer's Disease Neuroimaging Initiative Community-Science Partnership Board

Evolving from the original External Advisory Board, which consisted only of academic members, the ADNI CSPB was launched in June 2022 and includes both academic and community members. While the CSPB was launched during the DVTF efforts, the CSPB advised solely on ADNI4 efforts. Consistent with community-engaged research (CER) principles of co-learning and equity, it is co-chaired by four individuals: two community members (Bashir Easter and Sandra Talavera) and two scientists (the DVTF Co-Leads: O. Okonkwo and M. Rivera Mindt). The CSPB currently comprises seven community members who identify as Black, Latinx, and/or Asian (with care given to represent a variety of genders, socioeconomic levels, and geographic locations from across the U.S.) and six scientists from ADNI or other studies with expertise in brain health equity or CER (3 of whom identified as Black and/or Latinx). Representatives from the DVTF's marketing agency and additional ADNI team members (e.g., leaders from other Cores, project managers) served as *ex officio* members.

© 2024 Okonkwo OC et al. *JAMA Network Open.*

eMethods 6. ADNI3 inclusion efforts before the DVTF efforts.

Before the DVTF efforts, each site performed their own local recruitment and marketing efforts that were not overseen by the ADNI leadership and were not reported on. In addition to local site efforts, the ADNI3 Clinical Core worked with a marketing company on various nationwide and local digital marketing efforts which were not tailored to underrepresented populations. There were also small-scale efforts which used recruitment registries (e.g., Alzheimer's Prevention Registry) for outreach. There was no embedded infrastructure set up to track recruitment sources of enrolled participants.

© 2024 Okonkwo OC et al. *JAMA Network Open.*

eMethods 7. Participant characteristics metrics

Participant characteristics included self-reported race (American Indian/Alaskan Native, Asian, Black, Hawaiian/Other Pacific Islander, More than one, Unknown, White) and self-reported ethnicity (Hispanic/Latino, Not Hispanic/Latino), other sociodemographic characteristics [age (continuous), reported sex at birth (dichotomous), education in years (continuous), educational attainment groups (<=12 years, >12 years of education)], as well as diagnostic group (cognitively unimpaired, mild cognitive impairment, AD), and family history of AD/dementia. We created an “ethnocultural identity” variable which is mutually exclusive and based on self-identification, including Latinx (from any race), non-Latinx Black, non-Latinx Asian, and non-Latinx White. Due to the small number of participants from other ethnocultural groups, we created a variable called “Other non-Latinx Ethnocultural Group,” comprised of individuals self-reporting as American Indian/Alaska Native, Hawaiian/Pacific Islander, as well as those who indicated more than one race or declined to answer. It was necessary for this study to assess race and ethnicity since the main goal was to increase the enrollment of Latinx and Black older adults in the Alzheimer’s Disease Neuroimaging Initiative.

© 2024 Okonkwo OC et al. *JAMA Network Open.*

eTable 1. ADNI3 DVTF sites and Principal Investigators

Region	Site	City	Site Principal Investigators
**West**			
	Cleveland Clinic Lou Ruvo Center for Brain Health	Las Vegas, NV	Marwan N. Sabbagh
	Long Beach Veterans Administration	Long Beach, CA	Christopher Reist
	University of California – Los Angeles	Los Angeles, CA	Maryam Beigi
	Keck Medical Center of USC at University of Southern California	Los Angeles, CA	Lon S. Schneider
**Midwest**			
	Indiana University	Bloomington, IN	Martin Farlow
	Northwestern University	Chicago, IL	Emily Rogalski
	Rush University	Chicago, IL	Raj C. Shah
	Washington University	St. Louis, MO	Beau Ances
**Northeast**			
	Butler Hospital Memory & Aging Program	Providence, RI	Stephen Salloway
	Mount Sinai Hospital	New York, NY	Hillel Grossman
**Southeast**			
	Duke University	Durham, NC	P. Murali Doraiswamy
	Roper Hospital	Charleston, SC	Jacobo Mintzer
	Wien Center for Alzheimer's Disease and Memory Disorders at Mt. Sinai Medical Center	Miami, FL	Ranjan Duara

Note: The Principal Investigators listed above were current as of the launch of the DVTF (Diversity Taskforce)

© 2024 Okonkwo OC et al. *JAMA Network Open.*

eTable 2. Social media performance metrics

Performance metrics	Definition
Impressions	Total number of times ads were displayed
Reach	Number of unique individuals who saw the ads
Measure of ad engagement	Reactions, comments, saves, shares
Link clicks	Clicks to the microsite
Click-through rate	(Clicks/Impressions)*100

© 2024 Okonkwo OC et al. *JAMA Network Open.*

eTable 3. Counts of types of community-based organizations and types of outreach efforts

**Types of community-based organizations**	• Senior centers (n=4)<br>• Local memory and primary clinics (n=3)<br>• Churches (n=2)<br>• Health fairs (n=2)<br>• Community centers (n=1)<br>• Food pantries (n=1)
**Types of outreach efforts**	**Remote efforts**<br>• Local radio and TV (n=5)<br>• Webinars via Zoom (n=4)<br>• Newsletters (n=3)<br>• Newspapers (n=3)<br>• Social media posts (e.g., YouTube) (n=2)<br><br>**In-person presentations, booths and presence**<br>Location:<br>• Community-based organization (n=6)<br>• Community Event (n=3)<br>• Celebration (n=1)<br>• Health fair (n=2)<br>Mode:<br>• Presentations (n=9)<br>• Booth (n=2)<br>• Presence (n=2)<br><br>**Other in-person efforts**<br>• Memory screening days (n=1)<br>• Support groups (n=1)<br>• Caregiver training (n=1)<br>• Family referral (n=1)<br>• Referral from local individuals engaged in outreach (n=1)

© 2024 Okonkwo OC et al. *JAMA Network Open.*

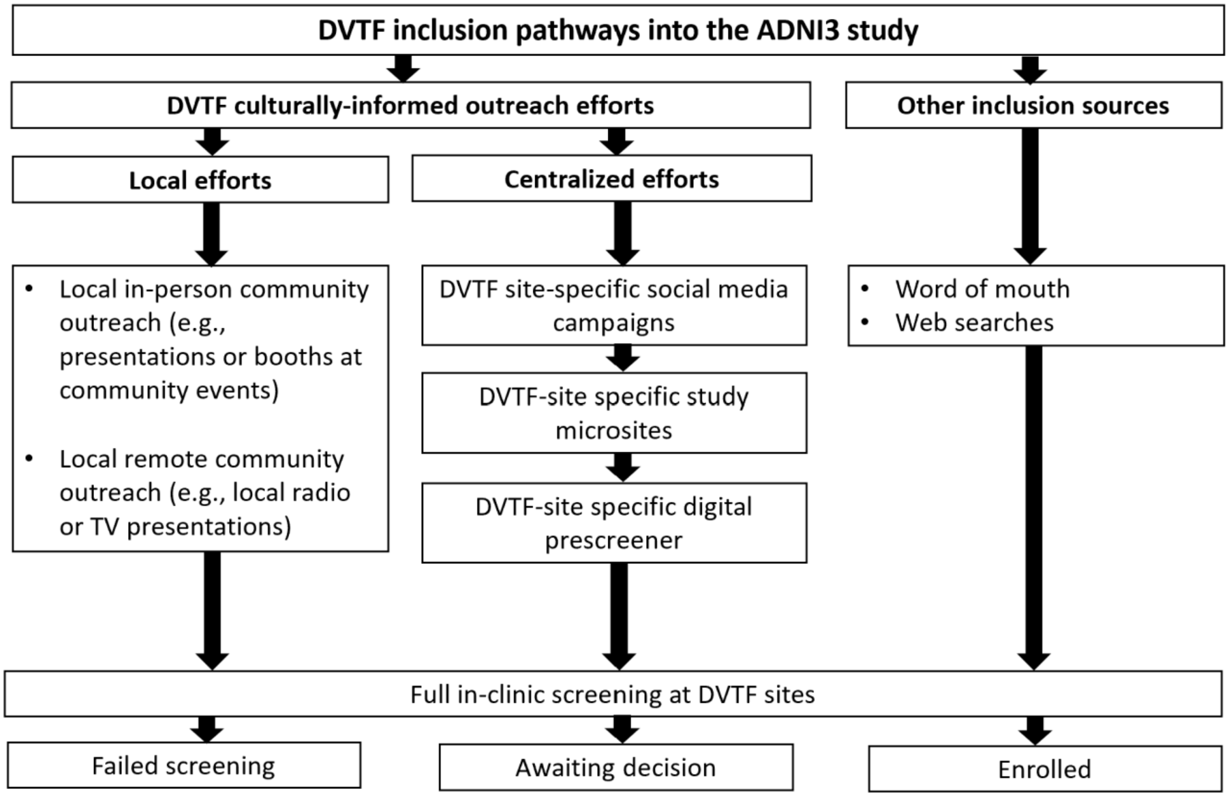

eFigure 1. Overview of the Diversity Taskforce inclusion pathways and efforts.

© 2024 Okonkwo OC et al. *JAMA Network Open.*

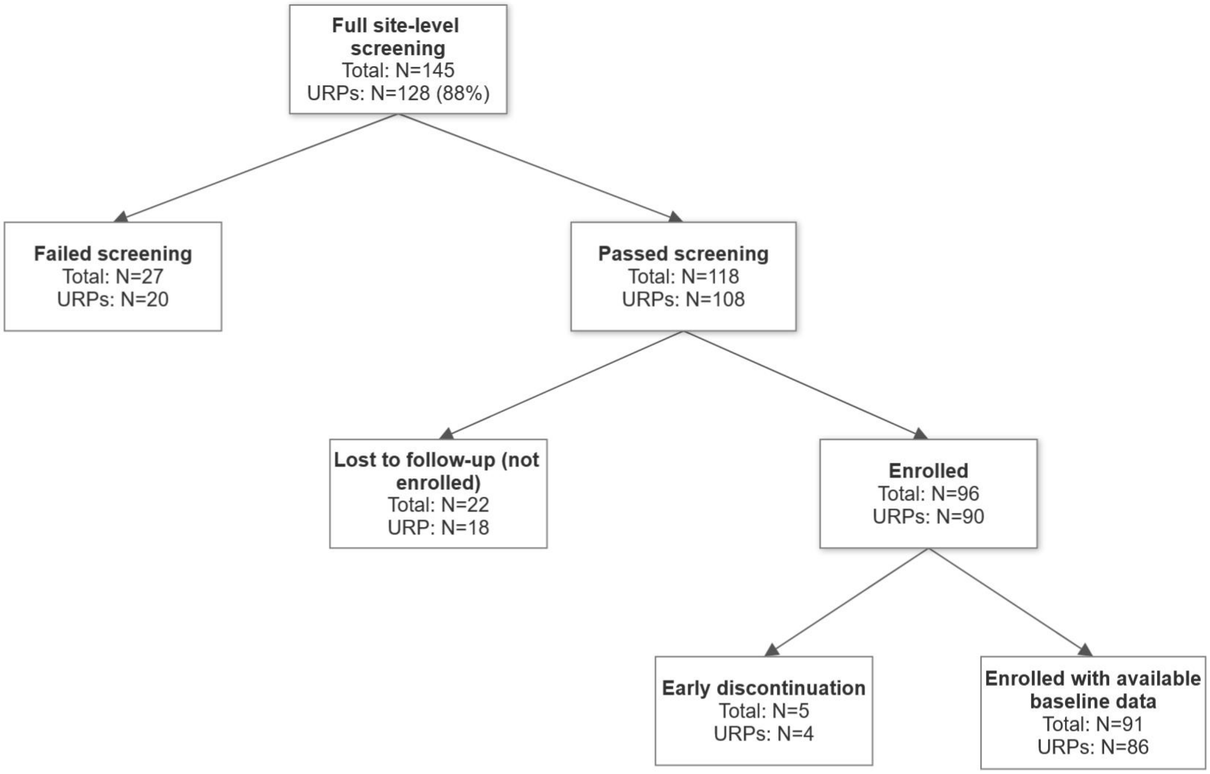

eFigure 2. Diversity Taskforce site screening and enrollment flowchart

Note: This flowchart is based on data retrieved from University of Southern California Laboratory of NeuroImaging (LONI).

© 2024 Okonkwo OC et al. *JAMA Network Open.*

eReferences

1. Weiner MW, Aisen PS, Jack Jr CR, et al. The Alzheimer's disease neuroimaging initiative: progress report and future plans. *Alzheimer's & Dementia.* 2010;6(3):202-211. e207.
2. Weiner MW, Veitch DP, Aisen PS, et al. The Alzheimer's Disease Neuroimaging Initiative 3: Continued innovation for clinical trial improvement. *Alzheimer's & Dementia.* 2017;13(5):561-571.
3. Weiner MW, Veitch DP, Aisen PS, et al. Impact of the Alzheimer's disease neuroimaging initiative, 2004 to 2014. *Alzheimer's & Dementia.* 2015;11(7):865-884.
4. Veitch DP, Weiner MW, Aisen PS, et al. Understanding disease progression and improving Alzheimer's disease clinical trials: Recent highlights from the Alzheimer's Disease Neuroimaging Initiative. *Alzheimer's & Dementia.* 2019;15(1):106-152.

© 2024 Okonkwo OC et al. *JAMA Network Open.*
