# Supplementary material for: A Protocol for the Inclusion of Minoritized Persons in Alzheimer Disease Research From the ADNI3 Diversity Taskforce
Source: JAMA Netw Open. 2024 Aug 9;7(8):e2427073. doi: 10.1001/jamanetworkopen.2024.27073 (PMC11316236; doi:10.1001/jamanetworkopen.2024.27073)
Supplement: Supplement 2. — Data Sharing Statement [file jamanetwopen-e2427073-s002.pdf]

Data Sharing Statement

Okonkwo. A Protocol for the Inclusion of Minoritized Persons in Alzheimer Disease Research From the ADNI3 Diversity Taskforce. *JAMA Netw Open*. Published August 09, 2024. doi:10.1001/jamanetworkopen.2024.27073

Data
Data available: Yes
Data types: Deidentified participant data
How to access data: All Alzheimer's Disease Neuroimaging Initiative (ADNI) data are shared without embargo through the LONI Image and Data Archive (IDA), a secure research data repository. Interested scientists may obtain access to ADNI imaging, clinical, genomic, and biomarker data for the purposes of scientific investigation, teaching, or planning clinical research studies. Access is contingent on adherence to the ADNI Data Use Agreement and the publications' policies outlined in the documents listed below. The application process includes acceptance of the Data Use Agreement and submission of an online application form. The application must include the investigator's institutional affiliation and the proposed uses of the ADNI data. ADNI data may not be used for commercial products or redistributed in any way. https://ida.loni.usc.edu/collaboration/access/appLicense.jsp
When available: With publication

Supporting Documents
Document types: None

Additional Information
Who can access the data: All data are shared without embargo through the LONI Image and Data Archive (IDA) Access is contingent on adherence to the ADNI Data Use Agreement and the publications' policies outlined in the documents listed below.
Types of analyses: Any purpose
Mechanisms of data availability: Acceptance of the Data Use Agreement and after approval of the online application.
Any additional restrictions: Data may not be used for commercial products or redistributed in any way.
